# Experimental Investigation on Ultrasonic Atomization Assisted Turning of Titanium Alloy

**DOI:** 10.3390/mi11020168

**Published:** 2020-02-05

**Authors:** Jianbing Meng, Bingqi Huang, Xiaojuan Dong, Yizhong Hu, Yugang Zhao, Xiuting Wei, Xiaosheng Luan

**Affiliations:** School of Mechanical Engineering, Shandong University of Technology, Zibo 255000, China; huangbingqi1995@163.com (B.H.); dongxiaojuan@sdut.edu.cn (X.D.); hyz576008000@163.com (Y.H.); zygsdut@126.com (Y.Z.); wxt@sdut.edu.cn (X.W.); lxs631781687@163.com (X.L.)

**Keywords:** titanium alloy, ultrasonic atomization assisted turning, tool wear, surface roughness, chip morphology

## Abstract

There are high cutting temperatures, large tool wear, and poor tool life in conventional machining, owing to the superior strength and low thermal conductivity of titanium alloy. In this work, ultrasonic atomization assisted turning (UAAT) of Ti6Al4V was performed with a mixed water-soluble oil-based cutting fluid, dispersed into tiny droplets by the high frequency vibration of a piezoelectric crystal. Different cutting speeds and two machining environments, dry and ultrasonic atomization assisted machining, were considered in the investigation of tool life, tool wear morphology, surface roughness, and chip morphology. In comparison with dry machining, UAAT shows lower tool wear and longer tool life due to the advantages of cooling and lubrication. Furthermore, better surface roughness, smoother chip edges, and shorter tool-chip contact length were obtained with UAAT.

## 1. Introduction

Titanium alloy has a high strength–weight ratio, good corrosion resistance, outstanding mechanical and thermal properties, and is currently one of the most widely used materials in aerospace, shipbuilding, medical, and other fields [1,2,3,4,5]. However, titanium alloy still has some shortcomings, such as low thermal conductivity, high strength, and easy chemical reactivity with tool materials, which impairs the machinability of conventional machining and makes it a typical difficult-to-cut material [6,7,8,9,10]. Dry cutting is a commonly used method in the green manufacturing of titanium alloys. Jiang et al. [11] conducted a comparative study between polycrystalline cubic boron nitride (PCBN) and a cemented carbide tool, and found that the PCBN tool had better durability in the high speed cutting of titanium alloy. Sun et al. [12] used the cutting tools of PCBN and polycrystalline diamond (PCD) in dry machining Ti6Al4V titanium alloy. They concluded that the strong diffusivity of the PCD material made the performance of the PCD tool better than that of the PCBN tool. Obviously, dry machining of titanium alloys shows high cutting temperatures, high tool wear, low tool life, poor surface quality, and a low material removal rate.

Laser-assisted machining (LAM) is an effective method, which reduces the cutting resistance of the material by locally heating and softening the workpiece [13]. Difficult-to-cut materials can be processed with a lower cutting force than that required by conventional machining. Compared with cooling tools, the softening of materials by laser heating can strengthen tool materials more effectively [14]. Ding et al. conducted a numerical modeling analysis in LAM for difficult-to-machine alloys and found that LAM can reduce or eliminate the formation of a built-up edge, and improve the surface quality in the micromachining process [15]. Elkhateeb et al. investigated the machining behavior of Ti6Al4V/TiC composites in LAM, and found that tool wear was improved by 68% [16]. Baek et al. compared the machining characteristics of AISI 1045 steel and Inconel 718 using conventional machining (CA) and LAM [17]. The experimental results showed that the cutting forces of AISI steel and Inconel 718 were reduced by up to 83% and 70%, respectively. Oh et al. investigated the machining characteristics of trochoidal milling and laser-assisted trochoidal milling of Ti6Al4V [18]. They found that the cutting forces decreases by approximately 33% to 41% during laser-assisted trochoidal milling.

Although LAM has already been used in various fields, there are still some drawbacks. Bejjani et al. found that an explanation of increased tool life with LAM has been related to tool–particle interactions, where the particles displace in the softer matrix instead of breaking and contributing to additional abrasion wear [19]. Additionally, LAM may increase surface roughness by up to 15%. Singh et al. carried out comparative studies on the cutting force, dimensional accuracy, and surface finish in LAM and conventional micromachining of H-13 mold steel [20]. The results indicated that the mean thrust force decreased by 17%, but the surface roughness increased by 36%. Jeon et al. pointed out that LAM increased the surface roughness and burr formation in micro end milling of aluminum and steel [21]. According to the above results, surface roughness and tool life are improved or decreased depending on machining conditions. Therefore, there is a significant need to develop some new methods to solve these problems in LAM.

Heat is generated at the cutting zone in LAM, and most of the resulting heat remains in the chip. Therefore, it is necessary to introduce a technique with cooling and lubricating effects to assist LAM. Although flood coolant and cryogenic coolant have noticeable abilities to reduce the heat at the cutting zone, they will worsen the softening effect of LAM. Minimum quantity lubrication (MQL) is considered as a potential alternative cooling strategy to conventional flood coolant and cryogenic coolant [22,23,24,25]. Bermingham et al. proposed a machining method by combining MQL with LAM. The hybrid method of laser and MQL improved tool life by suppressing the thermal wear processes while also preventing thermal fatigue on the cutting tool [26]. However, vegetable oils, mineral oils, and other oils selected as the base oil in MQL easily smoke and even burn in the hybrid process, owing to their lower ignition temperatures. Ultrasonic atomization (UA) is the application of mechanical vibrations of ultrasonic frequency on a thin liquid film, forming a capillary network on its surface, and a liquid drop at the top of the capillary wave can be separated by itself [27]. UA produces liquid droplets smaller than 100 nm, smaller than those produced by a spray nozzle of MQL [28]. This effect of UA could make the liquid droplets reach farther into the cutting zone and remove more cutting heat. Compared with MQL, UA coolant mainly uses water-based cutting fluids without smoke and combustion problems. Consequently, it is an attractive method to apply for LAM.

UA has been used for inhalation drug delivery, fuel combustion, analytical nebulizers, and air purification [29]. However, there is scarce literature or sufficient reported studies on the application of UA in machining [30]. This paper attempts to develop a novel aid for LAM and investigate the effects of ultrasonic atomization (UA) coolant with smaller liquid droplets on cutting performance. A mixed water-soluble oil-based cutting fluid was dispersed into tiny droplets by high frequency vibration of a piezoelectric crystal. The size of droplets was small enough to penetrate deep into the tool-workpiece interfaces, which significantly improved the interactive effect of cooling and lubrication. Experiments were performed to investigate the tool wear, surface roughness, and chip morphology in ultrasonic atomization assisted turning (UAAT) of Ti6Al4V alloy. For comparison, conventional dry machining was also conducted under the same cutting conditions.

## 2. Experimental Procedures

### 2.1. Experiment Conditions

**Figure 1:**
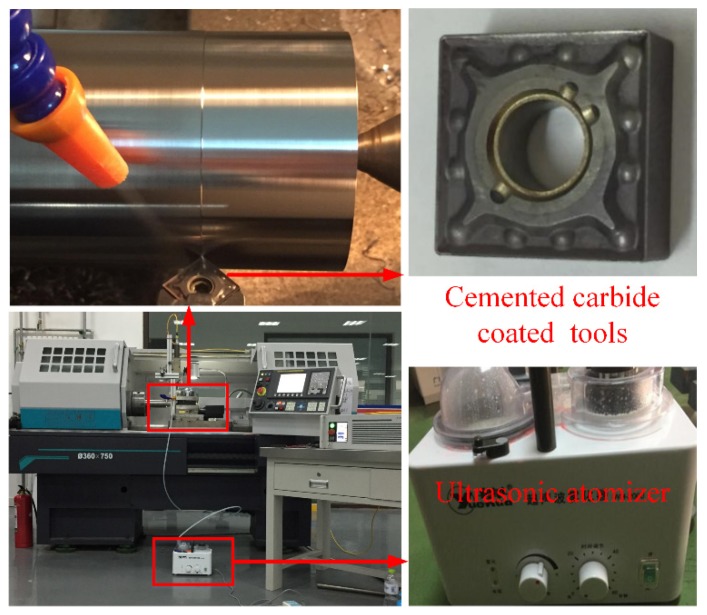
Experimental setup.

Ti6Al4V alloy was taken as the workpiece and prepared with a diameter of 60 mm and a length of 300 mm. Its chemical composition is given in Table 1, and the mechanical properties of Ti6Al4V are given in Table 2. *ρ*, *σ*_T_, *σ*_Y_, *δ*, *H*, *λ,* and *E* are the density, tensile strength, yield strength, elongation, hardness, thermal conductivity, and elastic modulus of Ti6Al4V, respectively. The experiments were carried out on CKD6136i made by Dalian machine tool Group Co. Ltd. (DMTG, Dalian, China). The schematic diagram of the computerized numerical control (CNC) turning lathe is shown in Figure 1. Its maximum spindle power is 5.5 KW, and the maximum diameter and length of the workpieces are 360 and 900 mm, respectively. In cutting tests of Ti6Al4V, cemented carbide turning inserts (SNMG120404-VP15TF, Jingchi Precision Machinery Co., Ltd., Qingdao, China) were used. The tool angles are shown in Table 3.

Water-soluble oil Huike 206 was dispersed into deionized water with fraction weight of 4 wt %. This type of mixing cutting fluid is environmentally friendly and suitable for industrial applications. An ultrasonic nebulizer (WH-2000, Yuehua Co., Ltd., Shantou, China) was used in this work, as shown in Figure 1. The ultrasonic frequency and power were 1.7 MHz and 80 W, respectively. It can be seen that the water generates an ultrasonic wave with the electronic oscillator circuit, which atomizes the cutting fluid into tiny droplets by the high frequency vibration of the piezoelectric crystal. The droplets were injected on the surface of the main cutting edge, near the cutting zone, to improve the cooling and lubricant effects.

### 2.2. Experiment Design

Two types of turning environments were considered, namely dry machining (DM) and ultrasonic atomization assisted machining (UAAM). The main turning parameters are the cutting speed, feed rate, and cutting depth, as shown in Table 4.

Scanning electron microscopy (SEM, Quanta 250 FEG, FEI Co., Ltd., Hillsboro, Oregon, USA) was used to capture the tool wear and chip morphology. The tool wear was measured three times, and the average value was regarded as the final tool wear value. The surface roughness was measured at three different positions of the workpiece after cutting distance *L*, using a portable roughness instrument (TR240, Beijing Times Co., Ltd., Beijing, China), and the average values were plotted. In addition, EDS of the tools was conducted using energy dispersive X-ray spectroscopy (EDS) spectrums. The cutting distance *L* of each group test can be calculated by the following Equation (1).

(1) $$L=v\frac{l}{n\cdot f}$$

where *v* is cutting speed (m/min), *l* is cutting length (mm), *n* is rotation rate (r/min), and *f* is feed rate (mm/r).

## 3. Results and Discussion

### 3.1. Tool Wear

Tool wear and its mechanism have been reported by Li and in other literature [31,32,33]. In this work, tool wear was evaluated on the basis of the wear of the tool flank. If the width of the flank wear reaches 0.3 mm (*VB* = 0.3 mm), it is regarded as the tool life criterion [34]. Two machining conditions, dry and ultrasonic atomization, were considered in the analysis of tool wear at different cutting speeds (30, 40, 50, and 60 m/min), with a constant cutting depth of 0.4 mm, and a feed of 0.1 mm/r.

**Figure 2:**
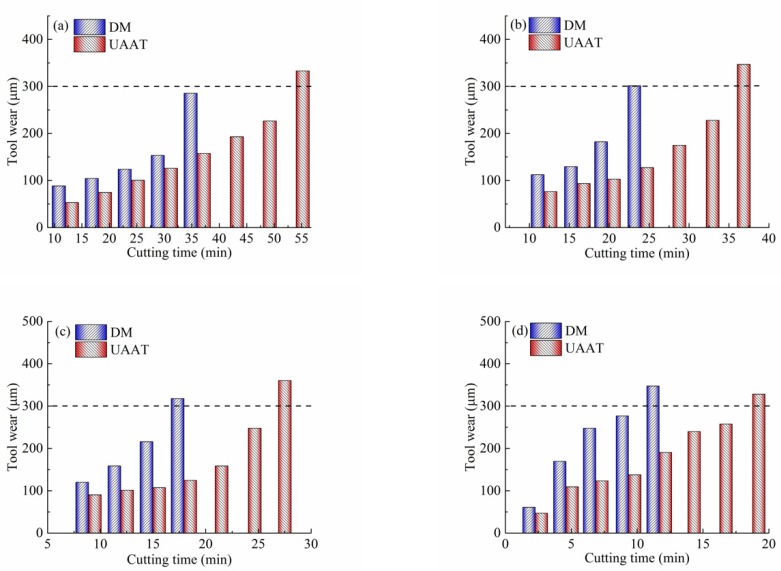
Tool life analysis at four cutting speeds: (**a**) 30 m/min, (**b**) 40 m/min, (**c**) 50 m/min, and (**d**) 60 m/min.

Figure 2 shows the influence of machining environments on the tool wear at different cutting speeds. As shown in Figure 2a, at a speed of 30 m/min, the tool life increased by 50% when the machining environment was changed from dry to ultrasonic atomization. A similar trend was observed at the medium cutting speeds of 40 and 50 m/min. Increases in tool life of 45% and 30% were measured if the machining condition changed to ultrasonic atomization, as shown in Figure 2b,c. Figure 2d shows the machining environment’s effects on the tool wear at the highest cutting speed of 60 m/min. Since a standard tool wear of 300 μm was used as the tool life criterion, the life of cutting tools under ultrasonic atomization assisted machining was approximately 1.6 times longer than that of dry machining. As shown in Figure 2d, a typical wear curve may be divided into three stages, which are the initial state, steady state, and accelerated state. An obvious increase of the tool wear rate was observed in the third state. However, it can be seen that ultrasonic atomization assisted turning (UAAT) presented decreased growth of tool wear. Thus, it is evident that UAAT has a positive impact on the reduction of cutting temperatures and frictional coefficients due to the cooling and lubricating effects of the droplets. In addition, from Figure 2, it can be seen that the tool flank wear growth rate was reduced by increasing the cutting speed.

**Figure 3:**
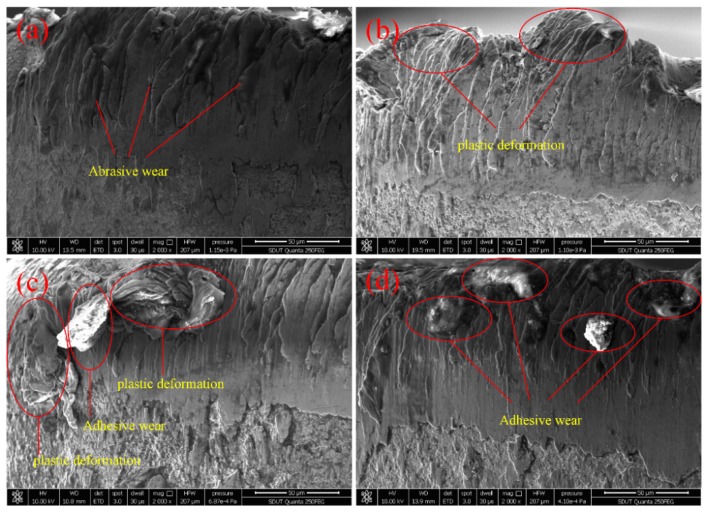
Tool morphology under dry machining at four cutting speeds: (**a**) 30 m/min, (**b**) 40 m/min, (**c**) 50 m/min, and (**d**) 60 m/min.

Figure 3 shows the tool morphology with the cutting distance *L* under dry cutting conditions. When the cutting speed is 30 m/min, the friction between the workpiece and the tool surface produces high temperatures. The tool coating falls off, owing to the different thermal expansion coefficients of the coating material and the tool substrate. Falling coating powders gather in the cutting area and result in obvious abrasive wear, as shown in Figure 3a. When the cutting speed increases to 40 m/min, a large amount of cutting heat cannot be released in time from the cutting area, resulting in the tool temperature rising too fast and forming a certain degree of plastic deformation, as shown in Figure 3b. Increasing the cutting speed, at the same time, results in the cutting length being greater than that of the low cutting speed. More cutting heat and friction are generated in the cutting zone, owing to the higher relative motion of the tool-workpiece interface and more material removal. When the cutting speed reaches 60 m/min, higher contact pressure and cutting temperatures are produced, due to the more violent friction between the workpiece and the tool flank. The Ti6Al4V workpiece is not difficult to bond onto the tool under high temperature and high pressure. Therefore, typical adhesive wear is produced, as shown in Figure 3b. It can be seen that tool wear under dry machining clearly shows a sticking and sliding zone. This means that the contact area consists of both a full metal-to-metal contact area and an abrasion area.

**Figure 4:**
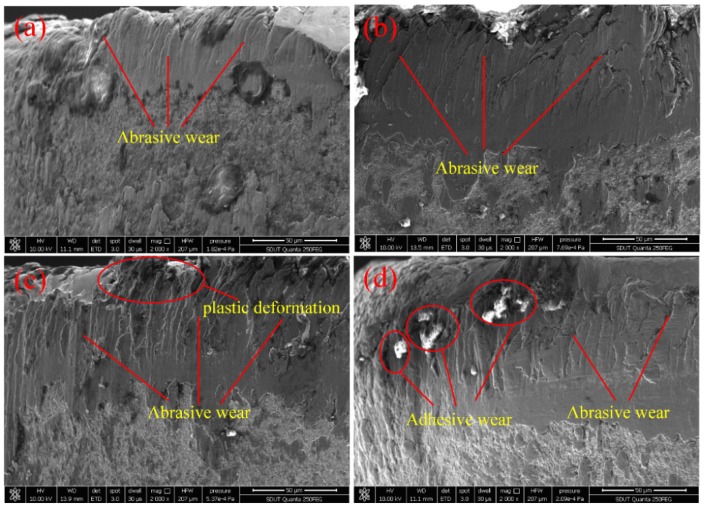
Tool morphology under ultrasonic atomization assisted machining at four cutting speeds: (**a**) 30 m/min, (**b**) 40 m/min, (**c**) 50 m/min, and (**d**) 60 m/min.

Figure 4 shows the tool morphology with the cutting distance *L* under UAAT conditions. When the cutting speed is 30 m/min, there is a relatively complete coating on the tool surface under ultrasonic atomization assisted cutting, as shown in Figure 4a. Even if the cutting speed increases to 40 m/min, the hardness of the coating material is still higher than that of the workpiece, due to the cooling effect of the atomized droplets. From Figure 4b, it can be observed that the lesser coating material falls off, which can reduce the abrasive wear of the tool. When the cutting speed increases to 50 m/min, the tool wear is mainly abrasive wear. Moreover, there exists a plastic deformation of the cutting tool, as shown in Figure 4c. Compared with dry cutting, the plastic deformation of the cutting tool is lesser under UAAT conditions. When the cutting speed reaches 60 m/min, more coating material falls off and adheres or welds to the tool flank, which causes a strong bond at the tool–workpiece interface. Therefore, typical adhesive wear is produced, as shown in Figure 4d. Compared with dry machining, the size of the adhesion and the amount of bonding in UAAT are smaller, which can decrease the cutting heat and temperature. The lower cutting temperature leads to a smaller cutting force. In addition, there is a mixture of tool wear mechanisms, such as adhesive wear and abrasive wear. Therefore, UAAT can significantly reduce or delay the adhesive wear of cutting tools, due to the cooling and lubricating effects of atomized droplets. Consequently, with the increase of the cutting speed, the tool wear in each cutting condition is characterized by abrasive wear, adhesive wear, and oxidation diffusion wear. However, when compared with the tool wear of dry machining, that of UAAT is smaller. In addition, the corresponding cutting speed of each stage of wear in UAAT is far less than that of dry machining.

**Figure 5:**
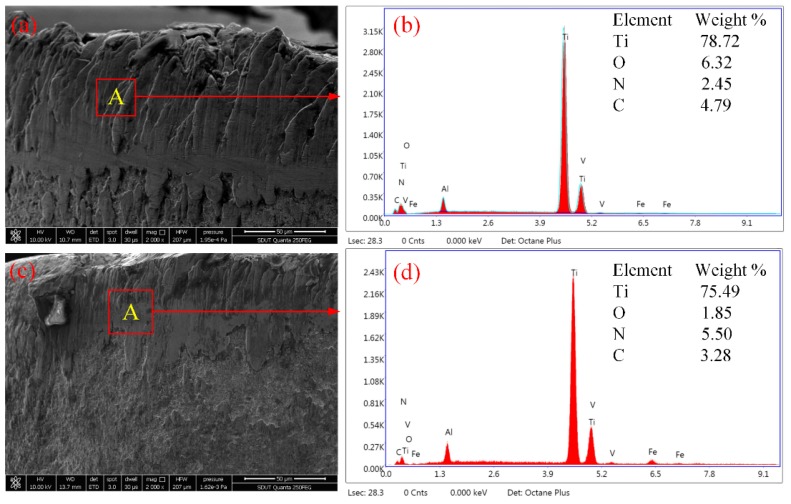
EDS spectra of Point A (**a**,**b**) under dry machining and (**c**,**d**) ultrasonic atomization assisted machining.

In order to characterize the mechanisms of tool wear, energy-dispersive X-ray spectroscopy (EDS) was taken from points, as shown in Figure 5a,c. The chemical composition of the points is given in Figure 5b,d. From Figure 5b, it can be seen that there were large amounts of titanium composition (78.72 wt %) at this point after dry machining. Thus, the point was an adhesive region of titanium, owing to the lower hardness and stronger affinity of the Ti6Al4V than the tool material under high-temperature and -pressure conditions. When adhesion occurs to a certain degree, stripping of the adhesive layer will happen to form the adhesive wear. Furthermore, the presence of the oxygen element (6.32 wt %) indicates that the oxidation wear occurred on the tool flank. It can be explained that titanium atoms in the tool coating spread outward after the outer coating rapidly stripped at a higher cutting temperature, and united with atmospheric oxygen atoms to form TiO_2_. According to Figure 5b, it is clear that the tool material contained some carbon elements, which were prone to unite with the titanium of the workpiece material and form a TiC layer. Hence, diffusive wear occurred under the combined effects of strong bonding in Ti6Al4V and a higher cutting temperature. Finally, it can be deduced that adhesive wear played a key role in the wear mechanism of the tool flank, and the occurrence of oxidation wear and diffusive wear should also be considered. Figure 5d shows the EDS spectra performed at Point A in Figure 5b. Compared with dry machining, the weight of oxygen decreased from 6.32 to 1.85 wt %, which resulted in a significant reduction of oxidation wear. In addition, the weight of titanium decreased by 3%. Thus, it is reasonable to say adhesive wear is reduced by UAAT, due to the cooling and lubricant effects of ultrasonic atomization.

### 3.2. Surface Roughness

**Figure 6:**
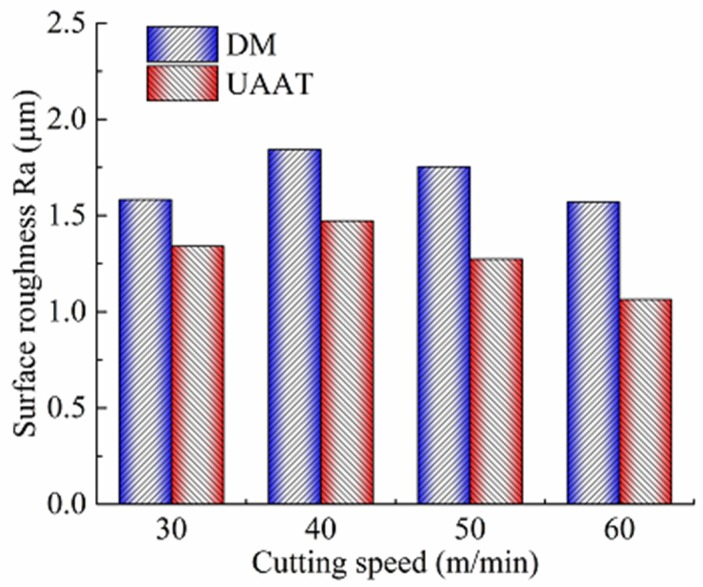
Surface roughness Ra with different cutting speeds under dry and ultrasonic atomization assisted machining conditions.

Figure 6 shows the comparison of surface roughness Ra with different cutting speeds at a feed of 0.1 mm/r and a cutting depth of 0.4 mm under two machining environments. Eight new cutting tools were used in dry machining and UAAT, with different speed of 30, 40, 50, and 60 m/min. Surface roughness was measured after the cutting distance *L* by each tool. Higher Ra was found under dry machining, owing to the higher adhesion and friction at the chip–tool interface. At the lower cutting speed of 30 m/min, the surface roughness Ra decreased by 15.2% when ultrasonic atomization was applied during machining. At the higher cutting speeds of 40 and 50 m/min, UAAT reduced the surface roughness by 20.1% and 27.4%, respectively. At the highest cutting speed of 60 m/min, a 47.6% decrease in the surface roughness was observed. Obviously, the surface roughness was improved by UAAT at different cutting speed. Increasing the cutting speed, the improvement of surface roughness by UAAT is more significant. Consequently, UAAT brings reduced surface roughness with the increase of the cooling, lubricant effect, and the reduction of tool wear.

### 3.3. Chip Morphology

**Figure 7:**
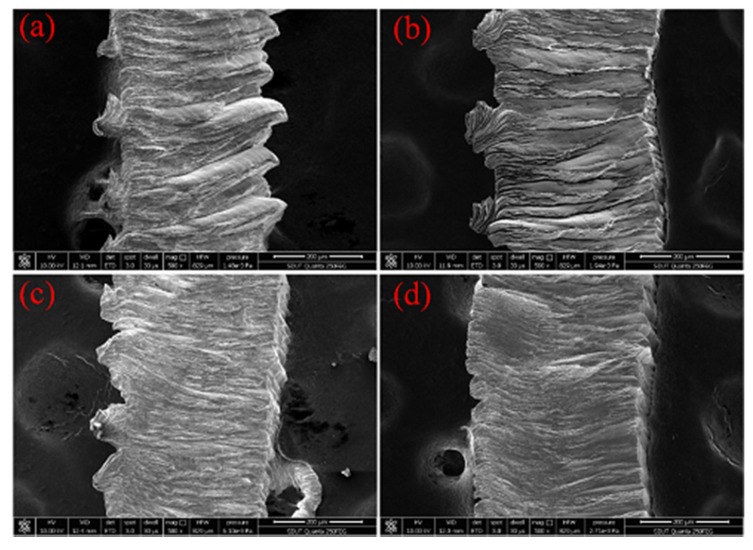
Chip morphology under (**a**,**b**) dry machining, and (**c**,**d**) ultrasonic atomization assisted machining at cutting speeds of (**a**,**c**) 20 m/min, and (**b**,**d**) 60 m/min.

Figure 7 shows the micrographs of chips produced by dry machining and ultrasonic atomization assisted machining. Dry machining exhibited low workpiece strength at a high cutting temperature. The curved chip formation can be found from Figure 7a,b, due to large deformation in the shear zone caused by the sharp cutting temperature. Under UAAT machining conditions, hard material could be cut due to the lower cutting temperature, and the curved chips produced by dry machining were replaced by straight chips. From Figure 7b,d, it can be seen that UAAT obtained smoother chip edges, due to the lubricant conditions between the chip and the cutting face. Lubricant effects resulted in a lower friction coefficient, and an increase in chip curling. As a result, the effective strain of deformed chips increased to the critical damage value, which led to the chip breakage. Furthermore, the smaller curvature radius of chip formation may be the main reason for reducing the tool–chip contact length under UAAT condition.

## 4. Conclusions

A novel process of ultrasonic atomization assisted turning (UAAT) was proposed for the machining of Ti6Al4V alloy. A series of turning experiments were conducted in order to investigate the effects of dry and ultrasonic atomization on the cutting performance. The tool life was characterized quantitatively, whereas the tool wear and the chip morphology were analyzed. The following conclusions can be drawn:

UAAT has a lubrication effect, which is useful to improve tool life. Under the same processing parameters, the tool life of UAAM was approximately 1.6 times longer than that of dry machining.

UAAT can reduce oxidation wear and adhesion wear. Under the same processing parameters, the content of elements such as oxygen and titanium decreased by 70.5% and 3%, respectively, compared with dry cutting.

UAAT has also a cooling effect, which helps to reduce the surface roughness of the workpiece. Under the same processing parameters, the surface roughness was reduced by up to 47.6% compared with dry machining.

Chip morphology revealed the formation of curved chips under dry machining and straight chips when ultrasonic atomization was employed. Shorter chips were produced by UAAT, with a smaller radius of curvature and smoother edges.

## Figures and Tables

**Table 1:** Chemical composition of Ti6Al4V.

Element	Ti	Al	V	Fe	O	C	N	H
Composition (wt %)	Balance	5.5–6.8	3.5–4.5	0.30	0.20	0.10	0.05	0.015

**Table 2:** Mechanical properties of Ti6Al4V.

Property	*ρ* (g/m^3^)	*σ*_T_ (MPa)	*σ*_Y_ (MPa)	*δ* (%)	*H* (HV)	*λ* (W/m∙K)	*E* (GPa)
Value	4430	990	830	14	312	7.9	114

**Table 3:** Tool angles.

Angle Types	Value (°)
Principal deflection angle	45
Auxiliary deflection angle	45
Rake angle	0
Back angle	0
Blade inclination angle	9

**Table 4:** Experimental parameters.

Machining Parameters	Value
Cutting speed (m/min)	30, 40, 50, 60
Feed rate (mm/r)	0.1
Cutting depth (mm)	0.4
Volume of output (mL/min)	2
